# Reciprocal regulation of nuclear factor kappa B and its inhibitor ZAS3 after peripheral nerve injury

**DOI:** 10.1186/1471-2202-7-4

**Published:** 2006-01-12

**Authors:** Lai-Chu Wu, Virginia M Goettl, Francesca Madiai, Kevin V Hackshaw, Syed-Rehan A Hussain

**Affiliations:** 1 Department of Molecular and Cellular Biochemistry, Ohio State University, OH 43210, USA; 2 Department of Internal Medicine, Ohio State University, OH 43210, USA; 3 Center for Molecular Neurobiology, Ohio State University, OH 43210, USA

## Abstract

**Background:**

NF-κB binds to the κB motif to regulate transcription of genes involved in growth, immunity and inflammation, and plays a pivotal role in the production of pro-inflammatory cytokines after nerve injuries. The zinc finger protein ZAS3 also binds to the κB or similar motif. In addition to competition for common DNA sites, *in vitro *experiments have shown that ZAS3 can inhibit NF-κB via the association with TRAF2 to inhibit the nuclear translocation of NF-κB. However, the physiological significance of the ZAS3-mediated inhibition of NF-κB has not been demonstrated. The purpose of this study is to characterize ZAS3 proteins in nervous tissues and to use spinal nerve ligation, a neuropathic pain model, to demonstrate a functional relationship between ZAS3 and NF-κB.

**Results:**

Immunohistochemical experiments show that ZAS3 is expressed in specific regions of the central and peripheral nervous system. Abundant ZAS3 expression is found in the trigeminal ganglion, hippocampal formation, dorsal root ganglia, and motoneurons. Low levels of ZAS3 expressions are also found in the cerebral cortex and in the grey matter of the spinal cord. In those nervous tissues, ZAS3 is expressed mainly in the cell bodies of neurons and astrocytes. Together with results of Western blot analyses, the data suggest that ZAS3 protein isoforms with differential cellular distribution are produced in a cell-specific manner. Further, neuropathic pain confirmed by persistent mechanical allodynia was manifested in rats seven days after L5 and L6 lumbar spinal nerve ligation. Changes in gene expression, including a decrease in ZAS3 and an increase in the p65 subunit of NF-κB were observed in dorsal root ganglion ipsilateral to the ligation when compared to the contralateral side.

**Conclusion:**

ZAS3 is expressed in nervous tissues involved in cognitive function and pain modulation. The down-regulation of ZAS3 after peripheral nerve injury may lead to activation of NF-κB, allowing Wallerian regeneration and induction of NF-κB-dependent gene expression, including pro-inflammatory cytokines. We propose that reciprocal changes in the expression of ZAS3 and NF-κB might generate neuropathic pain after peripheral nerve injury.

## Background

Peripheral nerve injury typically leads to multiple physiological alterations of the peripheral and central nervous system that includes changes in neuronal phenotype, increased excitability of spinal cord neurons, i.e., central sensitization, glial activation and disinhibition [1]. Collectively, these phenomena lead to the development and maintenance of neuropathic pain, through a complex web of signals and molecules that include inflammatory mediators at the site of injury, neurotransmitters, and chemokines at spinal cord synapses. Recent microarray experiments have further identified many genes that may contribute to neuropathic pain [2,3] Therefore, identifying the early events, namely the transcription factors, that trigger neuropathic pain may help to develop therapies to prevent or minimize the symptoms of this debilitating disease. Nerve injury induces production of pro-inflammatory cytokines, such as tumor necrosis factor alpha (TNFα), interleukin (IL)-1beta and IL-6, has been shown to play a key role in the propagation of neuropathic pain in animal models and human disease [4,5]. NF-κB is a key transcription factor that regulates the expression of those cytokine genes via the κB motif present in the promoters or enhancers [6-8] Consequently, NF-κB emerges as a potential drug target in the treatment of pathological pain [9-11]

The κB motif is a gene regulatory element controlling the expression of many genes involved in growth, immunity and inflammation. The regulation of κB-dependent transcription by the Rel family of NF-κB is well established [12,13] Recent studies, however, show that a family of large zinc finger proteins, called ZAS, also shares target genes with NF-κB [14]. Whereas NF-κB mostly induces transcription, ZAS proteins can positively [15-17] or negatively [18-20] regulate transcription. Additionally, a representative ZAS family member, ZAS3 (also known as Rc/KRC/HIVEP3), associates with an adaptor molecule in the TNFα signal transduction pathway, TNF receptor-associated factor 2 (TRAF2), to inhibit the nuclear translocation and transcriptional activity of NF-κB [20,21] Therefore, the interplay between ZAS3 and NF-κB may control important physiological processes, such as cell growth, apoptosis and cytokine expression.

RNA studies have shown that ZAS3 transcripts are expressed specifically in the lymphoid and nervous systems [22-24] Characterization of ZAS deficient mice suggests that ZAS2 and ZAS3 are involved in lymphoid development. There was a marked deficit in CD4(+)CD8(+) thymocytes in 6-month-old ZAS3(-/-);RAG2(-/-) chimeric mice, suggesting that ZAS3 is involved in T lymphocyte survival [25]. In addition, NF-κB is constitutively activated in ZAS3-deficient B cells, supporting the notion that ZAS3 associates with TRAF2 to inhibit NF-κB activation [20]. Similarly, in ZAS2-deficient mice, dramatic enhancement in the differentiation into T helper type 2 cells, constitutive activation of NF-κB, and enhanced GATA3 induction were observed [26]. Therefore, the ZAS proteins are able to inhibit NF-κB-driven transcription by competing with NF-κB for binding to DNA sequences similar to the κB motif and by inhibiting NF-κB activation [26]. However, the role of ZAS in the nervous system has not been studied. In the present work, ZAS3 proteins in nervous tissues have been characterized using two independent ZAS3 antisera. Abundant ZAS3 expression is found in neuronal cell bodies of the hippocampus formation, trigeminal ganglion, dorsal root ganglia (DRG), and motoneurons, and in astrocytes of the brain and spinal cord. Additionally, the down-regulation of ZAS3 and up-regulation of NF-κB in DRG after peripheral nerve injury suggests these κB-transcriptional proteins may be involved in neuronal regeneration and neuropathic pain.

## Results

**Figure 1:**
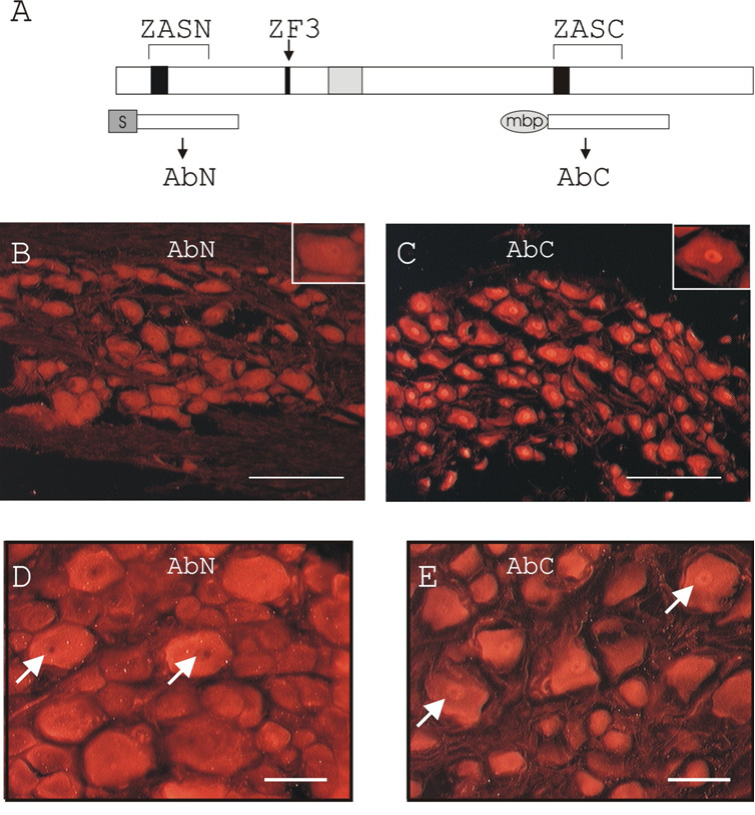
Primary structure of ZAS3 and expression of ZAS3 in the trigeminal ganglion and dorsal root ganglia. (A) Schema showing the domain structure of the ZAS3 protein and fusion proteins used to raise polyclonal antisera, AbN and AbC. *Black boxes*, zinc finger(s); grey box, middle conserved region; ZAS, zinc finger, acidic- and serine/threonine-rich regions; S, S protein; and mbp, maltose binding protein. (B) and (C) Immunohistochemical staining of sections of naïve rat trigeminal ganglion using antisera AbN and AbC, respectively. Inserts are higher magnifications showing uniform cellular staining of AbN and prominent nuclear staining with AbC. Bars represent 200 μM. (D) and (E) Longitudinal sections of DRG immunostained with AbN and AbC, respectively. Arrows highlight differential nuclear staining of AbN and AbC. Bars represent 100 μM.

The distribution of ZAS3 proteins in the rat nervous tissues was first determined by immunohistochemical procedures using ZAS3-specific antisera, AbN and AbC. Those antisera were raised against fusion proteins containing different vector encoded sequences and distinct regions of ZAS3 (Figure 1A). Each antiserum only interacted with the cognate antigens [17]. Consecutive rat coronal brain sections corresponded to the plane of bregma from -1 mm to -10 mm according to a stereotaxic atlas [27], longitudinal sections of isolated trigeminal ganglion, L3 to L6 DRG, and cross-sections of lumbar spinal cord were examined.

### ZAS3 expression in trigeminal ganglion

ZAS3-immunoreactivity (IR) was readily observed in the trigeminal ganglion, in agreement with abundant ZAS3 transcripts present in this tissue [24]. The signal intensity was strong from neuronal cell bodies and weak from nerve fibers (Figure 1B and 1C). Examination of individual ganglion nerve cell bodies under high power magnification revealed that signals derived from AbN were distributed uniformly between cytoplasm and nucleus (Figure 1B), whereas AbC yielded more intense signals from the nucleus (Figure 1C).

### ZAS3 in dorsal root ganglia

DRG was another nervous tissue where ZAS3-IR was readily observed (Figure 1D and 1E). Notably, AbN yielded intense signals mainly from large neurons, whereas AbC yielded similar signals from large, medium and small neurons. Similar to the immunostaining of the trigeminal ganglion, AbC yielded more prominent signals from the nuclei than from cytoplasm of DRG neurons, whereas signals from AbN were mostly cytoplasmic.

### ZAS3 in neurons and astrocytes of hippocampal formation and cerebral cortex

**Figure 2:**
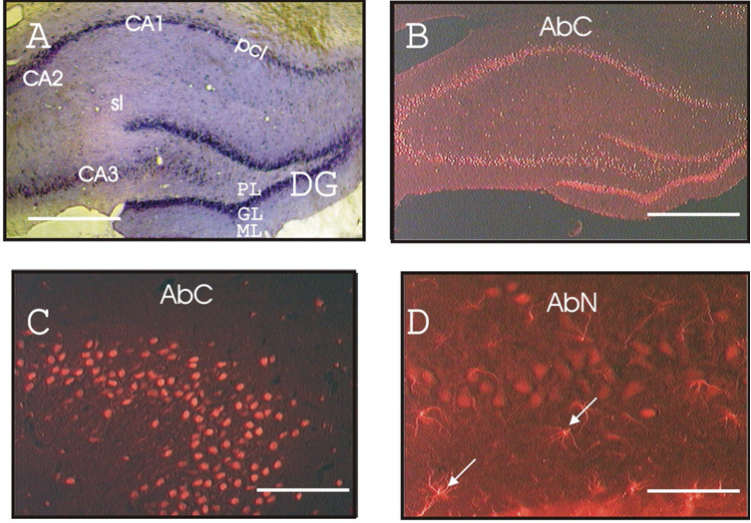
Expression of ZAS3 in neuronal cell bodies and astrocytes at the hippocampal formation. (A) Rat coronal brain section (Bregma -3.80) stained with cresyl violet; (B) immunostaining of a similar brain sections with AbC; (C) CA2 region stained with AbC; and (D) CA3 region stained with AbN. Star-like AbN-positive cells are highlighted with arrows. pcl, pyramidal cell layer; sl, stratum lucidum; PL, GL and ML, polymorphic, granule and molecular cell layer, respectively, of the dentate gyrus. Scale bars represent 2 mm (A and B); 0.2 mm (C); 0.1 mm (D).

Inspection of coronal sections through the septal half of the hippocampus showed abundant ZAS3-IR at the hippocampal formation (Figure 2). The expression of ZAS3 transcripts in the hippocampus of mouse embryos had not been seen in previous studies because para-sagittal sections were used for *in situ *hybridization [22,24] Neuronal cell bodies, including the pyramidal cells of the hippocampus proper Ammon's horn fields CA1, CA2 and CA3, and granule cells of the dentate gyrus, appeared as dark granules at low magnifications when stained with cresyl violet (Figure 2A). Cresyl violet reacted with the Nissl substance that was abundant in neuronal cell bodies. The pattern of ZAS3-IR in the hippocampal formation resembled that of cresyl violet in that both stained neuronal cell bodies (Figure 2B).

**Figure 3:**
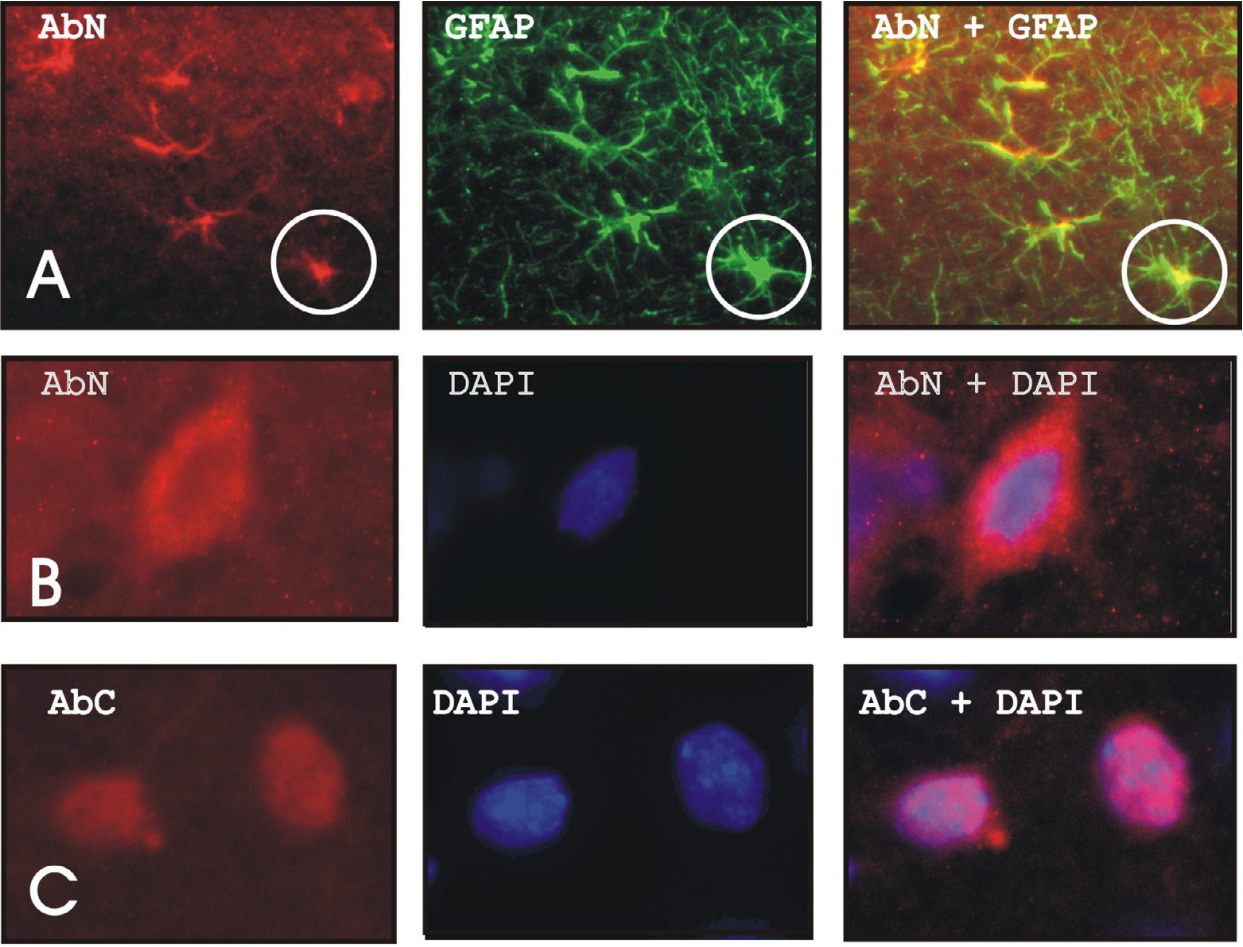
Expression of ZAS3 in astrocytes and neurons in the brain cortex. (A) Images of sections of brain cortex stained with AbN (left) and GFAP antibodies (middle). The images were superimposed in the right. Most AbN-IR cells were also stained by GFAP antibodies. Whereas AbN stained the cell bodies and the cylindrical processes, GFAP also stained the more elongated branching processes. An example of a cell stained with both AbN and GFAP antibodies is shown in a circle. (B) and (C) are high magnification of neurons stained with AbN, and AbC, respectively and DAPI. While AbC stained the nucleus, AbN yielded intense cytoplasmic staining.

The granule layer of the dentate gyrus consists mostly of neurons, whereas the pyramidal cell layers of the hippocampus have a larger glial population relative to neurons. In these hippocampus regions, AbC labeled only neurons (Figure 2C). On the other hand, AbN yielded modest labeling of neurons and more intense labeling of adjacent star-like cells that had elaborate processes (Figure 2D). Similar differential labeling pattern of both antibodies was observed in the cerebral cortex. Double labeling of the cortex with an antibody for the astroglial marker GFAP [28] confirmed that those AbN-positive cells were astrocytes (Figure 3A). Additionally, in neurons of hippocampus formation and cerebral cortex, AbN yielded stronger signals from the cytoplasm than from the nucleus (Figure 3B). On the other hand, the AbC signal was observed in nuclei but not in cytoplasm of neurons in the hippocampus (Figure 3C). Further, double labeling experiments with AbC and GFAP antibodies revealed that AbC only yielded signals from neurons but not from GFAP-positive cells. The ability of AbN but not AbC to react with astrocytes suggests that astrocytes may produce a ZAS3 isoform that does not contain the ZASC domain.

### ZAS3 in motoneurons

**Figure 4:**
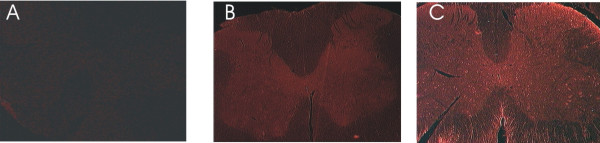
Expression of ZAS3 in the spinal cord. Cross section of rat L3 spinal cord stained with (A) pre-immune sera, (B) AbC, and (C) AbN. The expression of ZAS3 was higher in the grey matter than white matter.

**Figure 5:**
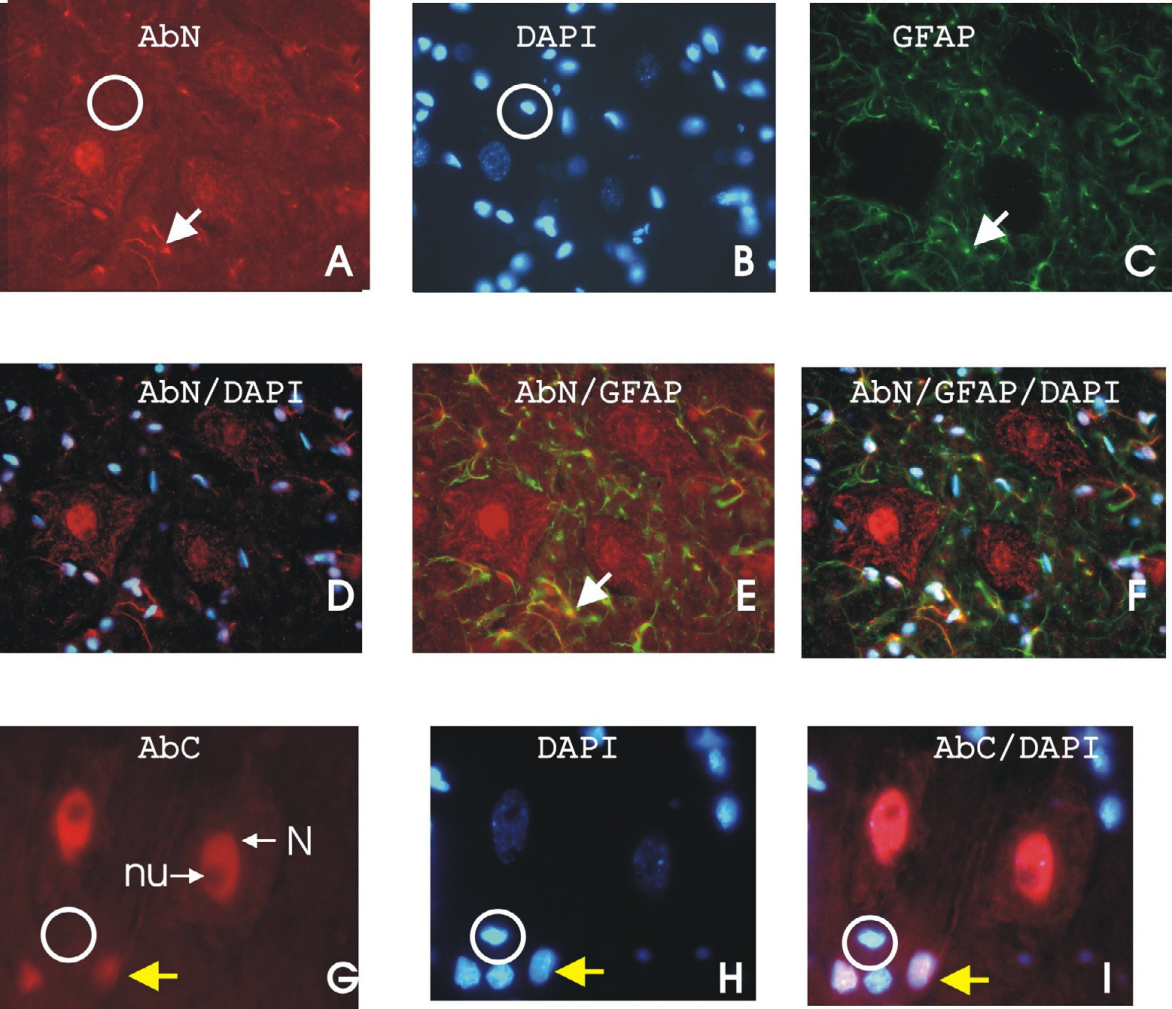
Expression of ZAS3 in motoneurons of lumbar spinal cord. Triple fluorescent ZAS3, GFAP, and DAPI staining of lumber spinal cord sections. Many cell nuclei were devoid of ZAS3 (circled), suggesting that the antisera, AbC and AbN, were specific. While AbC stained the nuclei of motoneurons, AbN stained both the nuclei and cytoplasm of motoneuorns and astrocytes. When the images of AbN and GFAP are superimposed, cells that expressed both proteins are yellow (highlighted with a white arrow in A, C, and E). When the images of AbC and DAPI are superimposed, nuclei expressing ZAS3 appear purple (highlighted with a yellow arrow), whereas nuclei that did not express ZAS3 are blue (circled).

The overall expression of ZAS3 was low in the lumbar spinal cord, where a more intense signal was observed in the grey matter than the white matter (Figure 4). Notably, abundant ZAS3-IR was observed in motoneurons in the ventral horn of the spinal cord (Figure 5). Double staining with ZAS3 antibodies and DAPI showed some cell nuclei that were devoid of ZAS3 (compared circled cells in Figure 5A and 5B for AbN and Figure 5G,H and 5I for AbC), showing that these antisera stained cells specifically. Again, AbC stained only the nuclei, whereas AbN stained both the nuclei and cytoplasm of motoneuorns. In addition, AbN also stained astrocytes, which were also positively labeling by antibodies against GFAP (such astrocytes were highlighted with white arrows in Figure 5A, 5C and 5E). As in the brain tissues examined, AbC stained only neurons in the spinal cord, whereas AbN stained both neurons and astrocytes.

The distribution of ZAS3 in various nervous tissues examined is summarized in Table 2. The similar tissue distribution of immunoreactivity derived from AbN and AbC, antibodies that were raised against distinct regions of ZAS3, suggests that these antibodies are specific for the endogenous proteins. In addition, AbC-IR is generally more abundant in the nucleus than cytoplasm, whereas the reverse is true for AbN. Furthermore, both ZAS3 antibodies reacted with neurons, whereas only AbN also reacted with GFAP-positive cells, which are astrocytes.

### Production of ZAS3 protein isoforms

**Figure 6:**
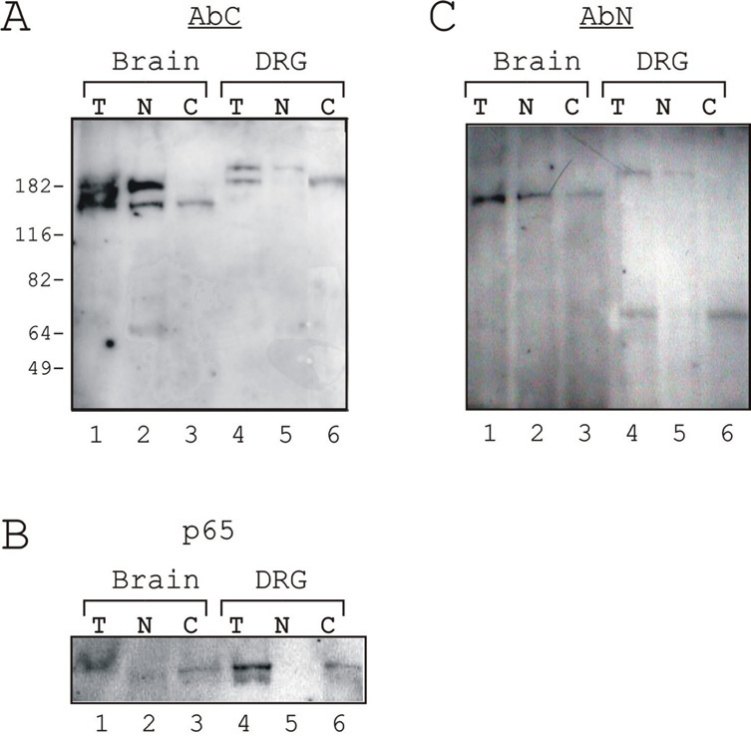
Immunoblot analysis of ZAS3 in brain and DRG. Proteins (T), cytoplasmic extracts (C), and nuclear extracts (N) resolved by SDS-PAGE was sequentially incubated with antibodies AbC (A), p65 (B), and AbN (C). T, total protein lysate; C, cytoplasmic extracts; and N, nuclear extracts.

Next, immunoblot analyses were performed to clarify the origin of the observed differences in the intensity of ZAS3-IRs between cytoplasm and nucleus. In total lysates of the brain, AbC reacted with two protein species of 190 kDa and 160 kDa in size (Figure 6A, lane 1). The smaller 160 kDa ZAS3 was present in both cytoplasmic and nuclear extracts, whereas the 190 kDa ZAS3 was observed only in the nuclear extracts (Figure 6A, lanes 2 and 3). AbC also reacted with two species in DRG, but with slightly retarded gel mobilities (Figure 6A, lane 4). The slower migrating species was found in the nuclear extracts, whereas the faster migrating species was present in the cytoplasmic extracts (Figure 6A, lanes 5 and 6). On the other hand, AbN reacted with a single 190 kDa species in the brain, but with two species of 200 kDa and 70 kDa in DRG (Figure 6C). In DRG, The 200 kDa species was observed in the nuclear extracts, whereas the 70 kDa protein was found in the cytoplasmic extracts. In general, the relative signal intensity observed from cytoplasmic extracts and nuclear extracts in immunoblotting was consistent with what we had observed in the immunohistochemical studies. The various species reacting with the ZAS3 antibodies suggest that ZAS3 isoforms were produced in a tissue-specific manner. As controls, equivalent loadings were visualized by protein staining before transferring proteins from gels onto nitrocellulose membranes, and p65-IRs were detected mainly from total protein lysates and cytoplasmic extracts, and minimally from nuclear extracts (Figure 6B). In most cell types, p65 associated with IκB and was therefore sequestered in the cytoplasm. The difference in gel mobility, immunogenicity, and cytoplasmic or nuclear distribution of ZAS3 isoforms suggest that the regulation of ZAS3 expression is complex and is different between brain and DRG.

### SpNL rats exhibit allodynia

A widely used peripheral neuropathy model, the L5/L6 spinal nerve ligation model (SpNL) [29] was used to examine whether ZAS3 may have a role in regenerative responses after peripheral nerve injury. Persistent allodynia, pain resulting from a non-painful stimulus, such as a light touch, is also a common characteristic of neuropathy. The behavioral changes of SpNL rats due to neuropathic pain after spinal nerve injury were confirmed by exhibition of mechanical allodynia in the left rear paw, i.e. the side ipsilateral to surgery, at day 6 post-operative. SpNL pre-surgery = 17.03 ± 2.89 g, post-surgery = 5.48 ± 3.32 g; p = 0.032 two-tailed Mann-Whitney non-parametric (n = 5). SHAM rats did not exhibit allodynia (pre-surgery = 23.11 ± 0.88 g; 6 days post-surgery 21.79 ± 2.2 g; n = 3); SpNL rats had maximum test values on the contralateral side 6 days post-surgery.

### In Silico cloning of rat ZAS3

**Figure 7:**
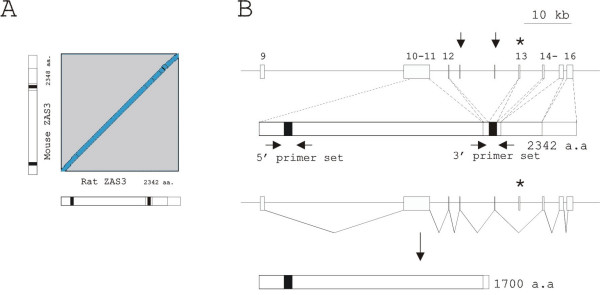
The rat ZAS3 gene and gene products. (A) A diagonal blot of the rat ZAS3 cDNA against mouse ZAS3 cDNA. (B) Schema of the rat ZAS3 gene (top panel), the complete protein product (middle panel), and a product generated by alternative splicing of exon 13 (bottom panel). White boxes represent exons; vertical arrows indicate two additional exons corresponded to a cDNA sequence present in GenBank databases (XM_233464); asterisk highlights exon 13 that is involved in alternative splicing; horizontal arrows mark the positions of primers used in RT-PCR; and black boxes represent zinc finger pairs. The 5' end of the rat ZAS3 gene has not been elucidated. Therefore, exon numberings, referring to the mouse gene, are shown on top of exons.

The relative amount of ZAS3 transcripts from ipsilateral and contralateral L5 and L6 DRG was compared by RT-PCR. Because rat ZAS3 had not been cloned, we first compiled an 8759 bp rat ZAS3 cDNA by *in silico *cloning procedure as described in Experimental procedures. The open reading frame of rat ZAS3 encodes a protein of 2342 amino acids, which is 92% identical to the mouse counterpart (Figure 7A). The cDNA was derived from 8 exons and covering a genomic region of 47 kb. Because the 5' end of the rat ZAS3 gene has not been characterized, the exon numbering of the mouse gene was used (Figure 7B). The 3' organization of the rat ZAS3 gene was largely similar to the mouse gene, and the exon and intron boundaries were also conserved. GenBank databases contained a shorter rat ZAS3 cDNA of 7440 bp (XM_233464), which was predicted by automated computational analysis of the gene sequence. Although XM_233464 was shorter, it encoded 2479 amino acids because it contained an insertion of 390 bp between exon 12 and exon 13. A comparison with the genomic sequence showed that those extra sequences were derived from two putative protein-coding exons (indicated with vertical arrows in Figure 7B). That 390 bp sequence, however, were not conserved in humans or mice, casting doubts onto its authenticity of being included in mature transcripts.

### Downregulation of ZAS transcripts in SpNL rats

Seven days post-surgery, L5 and L6 DRGs were analyzed for ZAS3 mRNA expression by semi-quantitative RT-PCR. Two primer sets were used to amplify distinct regions. The 5' region covers a 459 bp sequence, which corresponds to intron 10 of the mouse ZAS3 gene. Intron 10 is unusual in that when retained in transcripts it encodes the ZASN domain [30]. As in the mouse, the corresponding sequence in the rat starts with GC and ends with AG, which are splicing dinucleotides, allowing that 459 nucleotide sequence to serve as a potential intron. The 3' region contains exon 13, which is a 176 nucleotide sequence that encodes the carboxyl zinc finger pair of the ZASC domain. Those regions were chosen for amplification because they have been shown to be involved in alternative splicing in mouse (27). The alternative splicing of intron 10 will lead to the production of ZAS3 protein isoforms that lack the ZASN domain, whereas alternative splicing of exon 13 will lead to premature translation termination and production of carboxyl-truncated proteins that lack the ZASC domain [30].

**Figure 8:**
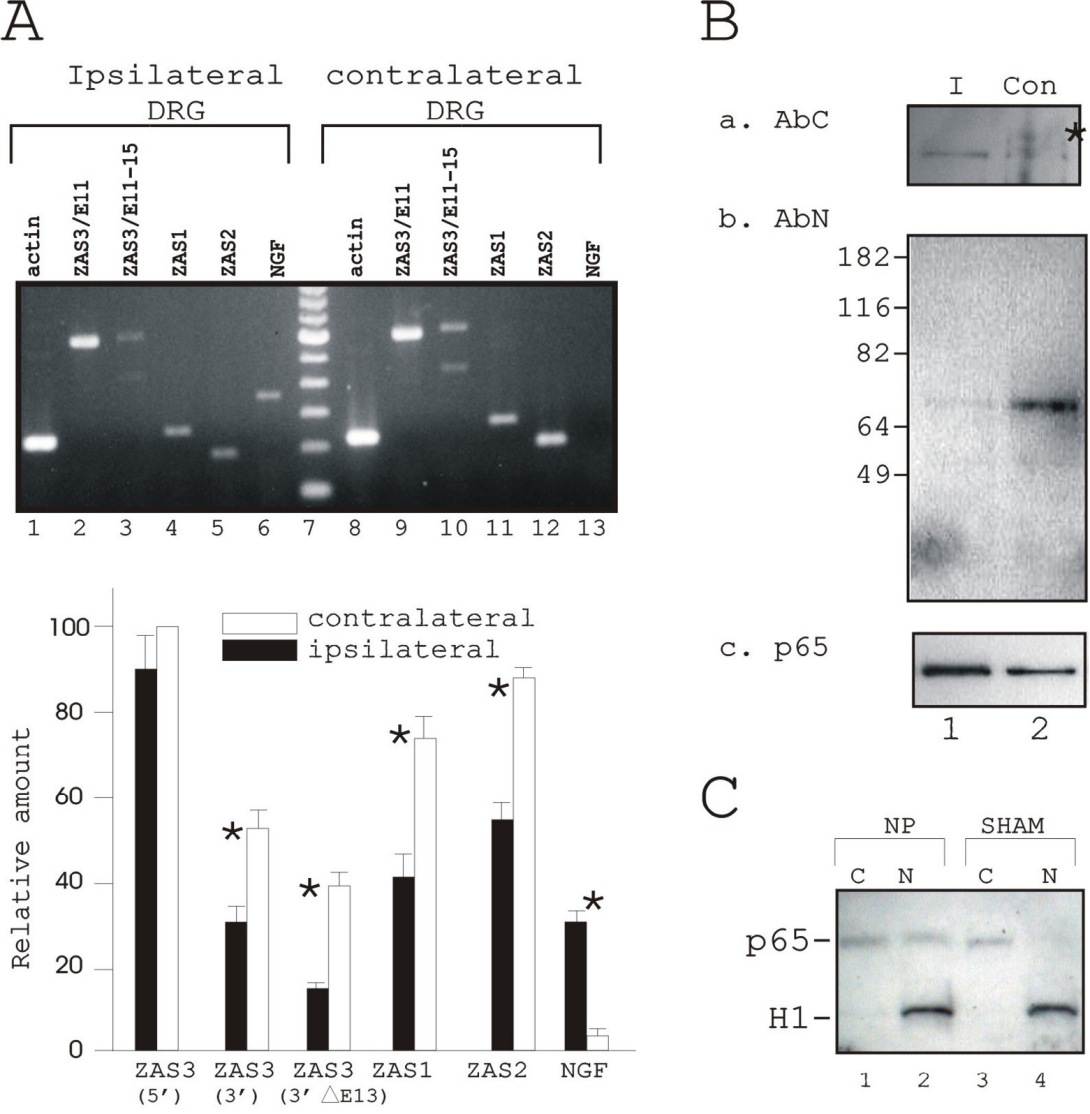
Downregulation of ZAS3 in SpNL. (A) Upper panels, RT-PCR transcript analyses of ZAS1, ZAS2, ZAS3, NGF and actin of DRG seven days after SpNL nerves (top panel). The experiments were carried out as described in the text (*n *= 4 per group) and a representative result is shown. (Bottom panel) Bar charts showing relative levels of each PCR products adjusted with actin in the same cDNA sample. Significance (*) is p < 0.05. (B) Immunoblot analyses of total protein lysates in ipsilateral (I) DRG and contralateral (Con) DRG prepare from rats seven days after surgery using AbC (a), AbN (b), and p65 antibodies (c). Asterisk indicates the 200 kDa species observed only in contralateral DRG but not in ipsilateral DRG. (C) Immunoblot analysis of cytoplasmic and nuclear extracts with p65 and histone H1 antibodies.

Seven days after SpNL surgery, ZAS3 mRNA levels were less in ipsilateral DRG than in contralateral DRG, suggesting that ZAS3 is downregulated in SpNL (Figure 8A). The formation of two PCR products of 601 bp and 422 bp using the 3' primer set showed that exon 13 of rat (Figure 8A, lanes 3 and 10), as in humans [31] and mice [30], was also involved in alternative splicing. Because the extra sequence in XM_233464 described in the above lies between exon 12 and exon 13 (Figure 7B), the lack of larger PCR products in this experiment supports our notion that the 390 bp sequence do not correspond to exons. On the other hand, alternative splicing of the ZASN region was not evident because only a PCR product of 588 bp, but no smaller products, was produced using the 5' primer set (Figure 8A, lanes 2 and 9). To determine whether downregulation in SpNL was specific for ZAS3 in the ZAS gene family, RT-PCR was also performed with ZAS1 and ZAS2 specific primer sets. ZAS1 and ZAS2 were also downregulated in DRG after SpNL. Nerve growth factor (NGF), whose expression was upregulated in SpNL [32], was used as a control for SpNL (Figure 8A, compare lane 6 and lane 13). Data from four independent experiments were summarized and presented in the lower panel of Figure 8A.

### Downregulation of ZAS3 proteins and upregulation of NF-κB in SpNL

Immunoblot analysis of total protein lysates revealed two species of 200 kDa and 190 KDa that reacted with AbC in contralateral DRG (Figure 8Ba, lane 2). In ipsilateral DRG, only the faster migrating species was observed (Figure 8Ba, lane 1). Similarly, the 70 kDa species, which reacted with AbN, was significantly diminished in ipsilateral DRG (Figure 8Bb, compare lane 1 and 2). Consistent with a decrease of ZAS3 and the inhibitory role of ZAS3 on NF-κB, the amount of p65 in the ipsilateral DRG was higher than that in the contralateral DRG (Figure 8Bc). Furthermore, the ratio of nuclear to cytoplasmic p65 in DRG of neuropathic rat was higher than that of SHAM rat (Figure 8C). The protein filter was also hybridized with antibodies against histone H1 as a control for nuclear extracts. Therefore, in SpNL ZAS3 was downregulated while NF-κB was upregulated.

## Discussion

This study localizes ZAS3 protein expression in neuronal cell bodies of the hippocampus proper and dentate gyrus (Figure 2), which contain groups of nerves that play a crucial role in the mechanisms of learning, emotion and memory. Abundant ZAS3 protein expression was also found in the trigeminal ganglion and DRG (Fig. 1). The trigeminal ganglion and DRG are equivalent structures, containing mostly neural crest-derived sensory neurons, which convey pain information from peripheral to central nervous system. DRG contain the spinal nerve's cell bodies and receive signals from the site of peripheral nerve injury to affect transcription and alter expression of neurotransmitters, receptors, and ion channels involved in modulating nociception. Additionally, ZAS3 expression is found in astrocytes in brain and spinal cord. Astrocytes are the major cell type in the brain and spinal cord whose function was previously thought to be limited to maintaining homeostasis for neuronal signaling [33]. Recently, we have shown increased astrocyte reactivity in the spinal cord after SpNL [34], and astrocyte activation is regarded as a driving force for creating and maintaining pathological pain states [35].

The expression pattern of ZAS3 in the nervous system prompted us to investigate whether ZAS3 plays a role in neuropathic pain, a pathological condition in which pain is evoked at much lower stimulus thresholds and often with greater severity. ZAS3 inhibits NF-κB, which regulates the transcription of proinflammatory cytokines, including IL-1, IL-6, and TNFα. Inflammation can lower nociceptor thresholds and increase neuronal excitability. We show for the first time that the expression of ZAS3 and its related genes, ZAS1 and ZAS2, is decreased in the ipsilateral versus contralateral L6 DRG seven days after SpNL (Figure 8). Rat DRG neurons survive and regenerate injured axons after nerve injury [36]. Because ZAS3 suppresses growth [25,37]. and NF-κB acts as a survival factor and promotes growth, the downregulation of ZAS3 and upregulation of NF-κB might be adaptations for sensory neuron mediated axonal regeneration after peripheral nerve injury.

It has been shown that injection of a NF-κB decoy, inhibitor for IkappaB kinase (IKK), or a TNFα antagonist to or near nerve lesions attenuated SpNL-induced mechanical allodynia or the upregulation of NF-κB-dependent genes [9,38]. In agreement with previous findings which showed that ZAS3 inhibits NF-κB [20], the downregulation of ZAS3 is in parallel with upregulation of NF-κB in SpNL. Therefore, the data suggest that a neuronal inflammatory cascade in DRG that involves ZAS3 and NF-κB may be important for the pathogenesis of neuropathic pain. The expression of ZAS3 in neurons of DRG and trigeminal ganglia, and in astrocytes, together with its reduction in DRG after SpNL suggest that ZAS3 may influence the genetic programming of sensory neurons and astrocytes, leading to neuropathic pain.

Immuno-studies show ZAS3 protein isoforms of 200 kDa, 190 kDa and 70 kDa are produced in DRG of naïve rats. The 200 kDa species, which reacted with both ZAS3 antisera, is located in the nucleus, whereas the 70 kDa species, which reacted with AbN but not AbC, is located in the cytoplasm (Figure 6). Likely, these ZAS3 protein isoforms may perform distinct functions in DRG. The size of the larger ZAS3 proteins and their immunogenicitysuggest it is most likely a ZAS3 protein isoform that is generated by an alternatively spliced ZAS3 transcript in which exon 13 was spliced out (Figure 7B, bottom panel). Alternative splicing of exon 13 is demonstrated by the smaller PCR product in RT-PCR using the 3' primer set (Figure 8A). ZAS3 transcripts that lack exon 13 will produce a truncated protein of 1700 amino acid residues that contains the ZASN domain, zinc finger 3 and the middle conserved region, but lacks the ZASC domain (Figure 7B, bottom panel).

The 70 kDa cytoplasmic ZAS3 protein isoform reacted with AbN but not with AbC. AbN was raised against a fusion protein that contained the ZASN domain (Figure 1A). In the mouse ZAS3 gene, the ZASN domain is encoded by intron 10 when it is retained in the mature transcript [30]. Exon 10-intron10-exon 11 together are more than 5.5 kb in size and encodes the first 1654 amino acids. Therefore, the 70 kDa ZAS3 protein isoform should be generated by an undefined alternative splicing event or by protein processing. Previously, it has been shown that this protein region associates with TRAF2 to inhibit TNFα/NF-κB signaling and with c-jun to induce AP1 transcription [21,39] Therefore, the cytoplasmic 70 kDa protein is an appropriate ZAS3 candidate isoform for the regulation of signal transduction.

As in most cell types, NF-κB is present mainly in the cytoplasm of unstimulated DRG neurons [40]. Immunoblot analyses show that in SpNL, the overall amount and nuclear localization of p65 in ipsilateral DRG was more than those in contralateral DRG (Figure 8). A similar increase and activation (that is nuclear localization) of p65-IR was observed in ipsilateral DRG neurons after tight ligation of the sciatic nerve [40]. Likely, upregulation and activation of NF-κB is a general response to peripheral nerve injury. In SpNL, downregulation of ZAS3 may lead to the translocation of NF-κB from the cytoplasm into the nucleus. NF-κB activation, and a release of κB-DNA binding competition and transcription repression by ZAS3 together in turn activates NF-κB-dependent gene expression, pro-inflammatory cytokine expression, sensitization of neurons, and neuropathic pain. Our observation suggests that after nerve injury, downregulation of ZAS3 may activate a cascade of NF-κB signal transduction and transcriptional events which leads to neuronal regeneration and neuropathic pain. Hence, via its regulatory role on NF-κB, ZAS3 might be a new target for the prevention, management, and resolution of persistent pain states following nerve injury.

## Conclusion

This report is the first to characterize the protein expression and function of a large zinc finger protein ZAS3 in the nervous system, and use an animal model to demonstrate a functional relationship between ZAS3 and NF-κB. Previous RNA studies have shown specific expression of ZAS3 in lymphoid and nervous tissues. Here immunohistochemical studies further localize ZAS3 expression to specific regions of the brain, including trigeminal ganglion and hippocampus formation, spinal cord, and dorsal root ganglia. Additionally, the cell-types that expressed ZAS3 in the nervous tissues are refined to neurons and astrocytes. The cellular distribution of the immunoreactivity and the results of Western blot analysis using cytoplasmic extracts and nuclear extracts suggest that ZAS3 protein isoforms with differential cellular localization are produced in a tissue-specific manner. The notion that ZAS3 may safe-guard cells from aberrant NF-κB activation stems from the fact that ZAS3 and NF-κB share similar DNA targets, the κB motif or similar sequences and that NF-κB is activated in ZAS3-knockout cells. *In vitro *studies further show that ZAS3 may inhibit the activation of NF-κB by associating with TRAF2. TRAF2 is an adaptor molecule essential for the assembly of a kinase complex that ultimately activates NF-κB. In this report, we further show the effect of L5 and L6 spinal nerve ligation (SpNL), a neuropathic pain model, on the expression of ZAS3 and NF-κB. In SpNL, ZAS3 transcripts and proteins are downregulated, whereas NF-κB is upregulated and activated. Thus, the reciprocal expression of ZAS3 and NF-κB in the neuropathic pain model described here provides a relevant physiological model to demonstrate the intimate functional relationship of these κB binding proteins, and suggest that downregulation of ZAS3 followed by upregulation of NF-κB to be two initial steps that triggers a cascade of gene activation and signaling events that lead to cytokine expression, and consequently, neuropathic pain.

## Methods

### Animals

Male Sprague-Dawley rats (Harlan, Indianapolis, IN) weighing 200 g to 250 g were used for all experiments in compliance with animal protocols approved by the Ohio State University Institutional Laboratory Animal Care and Use Committee. Each experiment or analysis was repeated with three to five animals, and consistent results were obtained.

### Spinal nerve ligation and testing for mechanical allodynia, and DRG isolation

For the spinal nerve ligation (SpNL) peripheral neuropathy model, rats were anesthetized with isoflurane and the left lumbar spinal nerves L5 and L6 were ligated tightly with 6-0 silk as described by Kim and Chung [29]. Sham surgery rats (SHAM) were treated similarly except that the spinal process was not removed to avoid damage to the underlying nerves, and the spinal nerves remained undisturbed. Allodynic responses to mechanical stimuli of both feet were determined using calibrated von Frey sensory monofilaments (Stoetling, Wood Dale, IL) and the "up and down" method [41] yielding a 50% threshold in g force. Rats were tested for two days before surgery, and at day 6 after surgery by an individual blinded as to the nature of the surgery. Post-surgery values are given in the text. Rats not responding to the stiffest fiber, 5.38, were given a score of 23.99 g. The Mann-Whitney non-parametric test was used to compare pre- and post-surgery ipsilateral SpNL allodynia. Behavioral values are expressed as mean ± S.E.M. Seven days post-surgery, rats were anesthetized with chloral hydrate, and the ipsilateral and contralateral L6 DRG were surgically removed, immediately frozen on dry ice, and stored at -80°C until analyzed.

### Immunohistochemistry

Naïve rats were deeply anesthetized with chloral hydrate (500 mg/kg), perfused transcardially with 200 ml of normal saline, and then with 500 ml of ice-cold 4% paraformaldehyde in 0.1 M phosphate buffer (pH 7.2). Whole brain, trigeminal ganglion, spinal cord, and L3 to L6 DRG were surgically removed and fresh frozen. Immunostaining of tissue sections (10–12 μM thick) was performed using polyclonal rabbit ZAS3 antisera, AbN and AbC, [17,42]., and goat glial fibrillary acidic protein (GFAP) antibodies (Santa Cruz, sc-6170) as described [34]. Some slides were also incubated with the fluorescent dye 4',6-diamidino-2-phenylindole (DAPI) (Molecular probe, D-1306; 0.5 μg/ml) to stain nuclei, or with cresyl violet (0.5%; ICN Biomedicals Inc., Aurora, OH) to stain neurons. Empirical experiments using a titration of each primary antibody from 100- to 1000-fold dilutions were performed to establish the best signal to background ratio. At least 6 slides from each tissue sample were examined, photographed using a Zeiss Axioscope fluorescent microscope attached to a digital camera (Magnafire Olympus), and consistent results were observed. Control experiments in which primary antibodies were replaced with normal rabbit serum or donkey serum yielded background signals. Controls for double label experiments consisted of incubating the slides with each primary antibody followed by the inappropriate secondary antibody.

### Immunoblot analyses

Brain, cut at Bregma -8.80 and the portion rostral to the rostral edge of the cerebellum, including most of the midbrain, all of the forebrain and the cortex but excluding the brain stem and cerebellum, and L5 and L6 DRG were used. Total protein lysates were obtained by homogenizing fresh frozen tissues in extraction buffer (5 ml/g tissues; 8 M urea and 4% CHAPS) with a pellet pestle and clearing by centrifugation. Cytoplasmic and nuclear extracts were prepared as described [25]. The presence of equivalent protein loading was shown by reversible protein staining (GelCode E-Zinc Reversible stain kit, Catalog number 24582; Pierce, Rockford, IL) of gels before transferring proteins onto nitrocellulose membranes. Immunoblot analyses using the ZAS3 antisera, antibodies against NF-κB p65 subunit, hsp90, or histone H1 (Santa Cruz Biotechnology) were performed as described [25].

### In Silico cloning of rat ZAS3 cDNA

The published mouse ZAS3 cDNA (8755 bp; GenBank Accession Number L46815) was input for BLAST analysis against the rat genomic DNA sequences (NW_047719) in the NCBI databases. Exons, predicted from matching rat gene sequences and referred to the mouse exon intron boundaries (23), were joined to obtain a rat ZAS3 cDNA sequence of 8759 bp.

### Reverse transcriptase-polymerase chain reaction

Total RNA was isolated from fresh frozen DRG using the Purescript total RNA kit (Gentra Systems, Minneapolis, MN). Semi-quantitative RT-PCR was performed with total RNA using a one-step RT-PCR kit (catalog number 10928-034; Invitrogen Carlsbad, CA). The sequences of each gene-specific primer set used, GenBank accession numbers, and size of PCR products are shown in Table 1. Synthesis of cDNA was performed at 50°C for 20 min. PCR conditions were 94°C for 1 min; 56°C for 1 min; 72°C for 45 sec (29 cycles); and a final extension step at 72°C for 10 min. These conditions gave linear amplification of all mRNA examined. PCR products were resolved by agarose gel electrophoresis and visualized by ethidium bromide staining. The relative amount of each PCR product was determined from photographs of DNA gels using LabWorks™ Image Acquisition and analyzing Software (UVP Inc. Upland CA 91786) and normalized to β-actin. The values between ipsilateral and contralateral sides were analyzed by Student's t-test, and when p < 0.05, the data were considered statistically significant.

## List of abbreviations used

ZAS zinc finger, acidic and ser/thr-rich regions

κB kappa B

NF-κB nuclear factor kappa B

TNFα tumor necrosis factor alphpa

TRAF2 tumor necrosis factor receptor-associated factor 2

IL interleukin

DRG dorsal root ganglia

HIVEP3 human immunodeficiency virus type 1 enhancer-binding protein 3

SpNL spinal nerve ligation

RT-PCR reverse transcription-polymerase chain reaction

## Authors' contributions

LCW performed Western blot analyses, analyzed DNA sequences, prepared the figures and wrote the draft of the paper. VMG maintained rats, performed the spinal nerve ligations, conducted mechanical allodynia test, and performed statistical analyses. FM isolated the tissues and performed immunohistochemical experiments. KVH initiated the neuropathic pain study. SRAH helped isolated the tissues and prepare RNA to perform the quantitative RT-PCR.

## Figures and Tables

**Table 1:** Gene-specific primer sets for RT-PCR

Gene	forward primer/reversed primer	PCR product size (bp)
NGF	481 TCATCCACCCACCCAGTC 498 834 TCTTGCAGCCTTCCTGCT 817	354
β-actin	382 CACCCTGTGCTGCTCAC 398 598 GAGGGAGCGCGTAACCCT 581	217
ZAS1	6832 ACTCCCATCTCCCCAGAAGT 6851 7081 ACGGTTGCAAGGTTATTTGC 7062	250
ZAS2	7211 ATGGGGCAGTATCTGCAAAG 7229 7395 TGAGAATGGAGGGGAAGATG 7376	185
ZAS3	1693 CCCTCACAGGTCTCCCTGAA 1712 2280 GGCCAGCTTCTGCTTGATGG 2261	588 138
ZAS3	6056 TCCTGAGATCTAAGCAGAAG 6075 6665 GAGTCTGAGTCAGAGTCCTC 6646	601 422

**Table 2:** Tissue and cellular distribution of ZAS3-IRs

Tissues	ZAS3 antibodies AbN	ZAS3 antibodies AbC
Hippocampus formation		
Neurons	+ (cyto > nuc)	++ (nuc)
astrocytes	++	-
Trigeminal ganglion		
Neurons	++ (cyto ~ nuc)	++ (nuc > cyto)
Cerebral cortex		
Neurons	+ (cyto > nuc)	++ (nuc)
Astrocytes	++	-
DRG		
Neurons	++ (cyto > nuc)	++ (nuc > cyto)
Lumbar spinal cord		
Motoneurons	++ (cyto ~ nuc)	++ (nuc)
astrocytes	++	-

background signals (-); relative signal intensity is reflected by the number of (+); cyto, cytoplasm; and nuc, nucleus.
